# Combined Effects of Mating Disruption, Insecticides, and the Sterile Insect Technique on *Cydia pomonella* in New Zealand

**DOI:** 10.3390/insects11120837

**Published:** 2020-11-27

**Authors:** Rachael M. Horner, Peter L. Lo, David J. Rogers, James T. S. Walker, David Maxwell Suckling

**Affiliations:** 1 The New Zealand Institute for Plant and Food Research Limited, Private Bag 4704, Christchurch 8140, New Zealand; Max.Suckling@plantandfood.co.nz; 2 The New Zealand Institute for Plant and Food Research Limited, Havelock North 4157, New Zealand; Peter.Lo@plantandfood.co.nz (P.L.L.); Dave.Rogers@plantandfood.co.nz (D.J.R.); jim.walker@plantandfood.co.nz (J.T.S.W.); 3 School of Biological Sciences, University of Auckland, Auckland 1072, New Zealand

**Keywords:** sterile insect technique, eradication, suppression, orchard, biosecurity, *Cydia pomonella*, Lepidoptera, Tortricidae, Unmanned Aerial Vehicle, market access, mating disruption, synergistic

## Abstract

**Simple Summary:**

Codling moth is a major pest of apples, and was accidentally introduced into New Zealand over 150 years ago. Many countries that New Zealand exports apples to do not have codling moth present and they want to keep it out. Therefore, apple growers must heavily control codling moth populations on their orchards. Currently, the main control tactics are insecticide applications and mating disruption, which uses the moth’s own sex pheromone to make the males unable to find females to mate. We aimed to supplement these tactics with the sterile insect technique (SIT) to further suppress the codling moth on orchards. SIT involves mass rearing and sterilizing codling moth and then releasing them onto orchards where they mate with wild insects resulting in no offspring. We released sterile insects onto seven orchards using unmanned aerial vehicles and ground releases. Six years of the program saw significant drops (90–99%) in wild moth populations. The SIT is an excellent tactic for reducing moth populations in export apple orchards.

**Abstract:**

Codling moth was introduced into New Zealand, and remains a critical pest for the apple industry. Apples exported to some markets require strict phytosanitary measures to eliminate the risk of larval infestation. Mating disruption and insecticide applications are the principal means of suppression in New Zealand. We tested the potential for the sterile insect technique (SIT) to supplement these measures to achieve local eradication or suppression of this pest. SIT was trialed in an isolated group of six integrated fruit production (IFP) orchards and one organic orchard (total 391 ha), using sterilized insects imported from Canada, with release by unmanned aerial vehicle and from the ground. Eradication was not achieved across the region, but a very high level of codling moth suppression was achieved at individual orchards after the introduction of sterile moths in combination with mating disruption and larvicides. After six years of releases, catches of wild codling moths at three IFP orchards (224 ha) were 90–99% lower than in 2013–2014, the year before releases began. Catches at three other IFP orchards (129 ha) decreased by 67–97% from the year before releases began (2015–2016), from lower initial levels. At a certified organic orchard with a higher initial population under only organic larvicides and mating disruption, by 2019–2020, there was an 81% reduction in wild moths capture from 2016–2017, the year before releases began.

## 1. Introduction

Codling moth (*Cydia pomonella*) L. is a key pest of apple, and is native to Central Asia, but has spread to most temperate apple growing regions including North America, Australia, South Africa, Europe, and China [1]. It was accidentally introduced to New Zealand over 150 years ago [2] and is only known to have been eradicated from Western Australia [3]. An earlier era of single tactic control of codling moth by organochlorine insecticides in the 1950s and later organophosphate insecticides required to achieve phytosanitary standards for export led to problems with insecticide resistance [4,5], reduced biodiversity [6], and unwanted residues on fruit [7]. Fumigation of fruit with methyl bromide was also developed as a phytosanitary treatment for certain markets [8]. Concern over the lack of sustainability of these technologies led to research and development of alternatives such as sex pheromones for apple pests [9,10]. The adoption of these technologies along with the enhancement of existing and new classical biological control agents and selective insecticides has led to the development and wide adoption of more sustainable apple pest management and production systems [11]. In New Zealand, the established codling moth parasitoid fauna is considered to be limited to *Liotryphon caudataus*, *Glabridorsum stokesii, Mastrus ridens* (Hymenoptera: Ichneumonidae), pupal ectoparasitoid *Dibrachys microgastri* (Hymenoptera: Pteromalidae), and the egg-larval parasitoid *Ascogaster quadridentata* (Hymenoptera: Braconidae). There is limited contribution to control of codling moth in New Zealand from these introduced natural enemies [12,13]. This may be because of the low tolerance by orchardists for their codling moth host, resulting in a very low density of specific natural enemies in most orchards [14]. This suggests that unmanaged codling moth populations should be present in higher densities than in export orchards, even in the presence of biological control agents, which appears to be the case [15].

**Figure 1:**
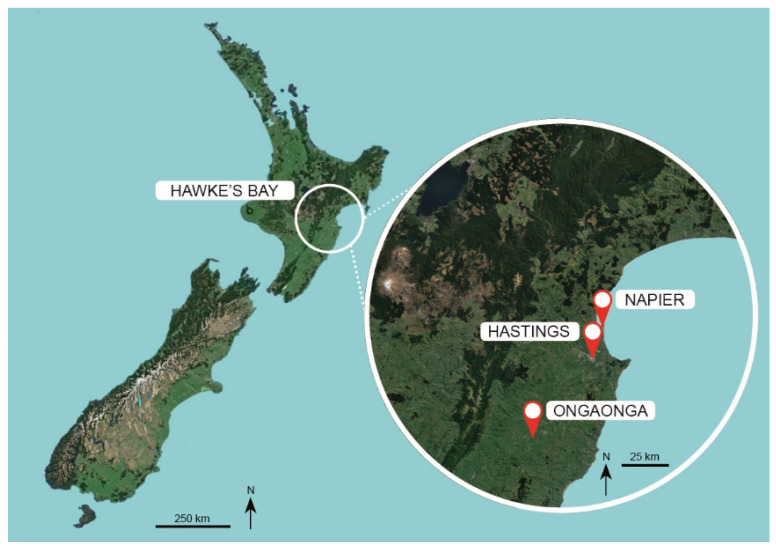
Location of the pilot codling moth (*Cydia pomonella*) sterile insect technique (SIT) program near Ongaonga in Central Hawke’s Bay, New Zealand, ~45 km from the main orchard district around Hastings, Hawke’s Bay. Map image courtesy of Google Earth (4 May 2020).

New Zealand had 10,179 ha of planted apples and exported 383,500 t of apples to 75 countries in 2019 [16], with most apples grown in Hawke’s Bay (Figure 1). In 2019, apple exports were worth US$ 538 million, with US$ 216 million derived from exports to Asia [17]. Codling moth is a quarantine pest for many Asian markets, consequently they require strict phytosanitary measures to eliminate the risk of larval infestation associated with imported fruit. Export apple growers follow integrated fruit production (IFP) principles [11], including a shift from calendar schedules of insecticides and fungicides to justified use, based on pest and disease monitoring systems and applications of new selective insecticides. The industry adheres to the New Zealand Ministry for Primary Industries’ ‘systems-based’ program, where codling moth risk is managed along the entire production pathway: production sites are registered, there are systems of pheromone traps, crops are treated when a threshold is reached, crop protection inputs are independently audited, and there is traceability of the crop and all inputs. Despite the high phytosanitary performance of this regulatory program, certain high-value markets have a zero tolerance policy for live codling moth in fruit and require mandatory postharvest disinfestation with the fumigant methyl bromide. A dynamic situation exists, with New Zealand aiming to eliminate or recapture any emissions of this ozone-depleting gas at a national level [18], which has justified a recent classical biological control introduction of *Mastrus ridens* [13]. However, the parasitism rate by *Mastrus ridens* of sentinel codling moth larvae in Hawkes Bay was as low as 1% [13].

Adoption of the concept of area-wide control has been promoted within New Zealand apple production, whereby pest populations can be contained at low levels for longer periods and pest management methods can be integrated that are less reliant on pesticides and that better address ecological and environmental concerns [7,11,19]. At the same time, economic units have increased in size and corporate apple orchards have emerged with many hundreds of hectares under unitary management. Export-focused and documented suppression of codling moth has involved submission of electronic spray diaries to the Ministry for Primary Industries and insect counts at the landscape scale, and a deliberate industry-wide shift towards more benign control tactics to reduce and ultimately avoid residues [11]. Mating disruption in particular has dramatically reduced Hawke’s Bay orchards’ populations of codling moth [20], yet codling moth remains a problem, because adoption of this technology remains around 35% [11]. In addition, substantial peri-urban codling moth populations are likely re-seeding some orchards [15]. Combinations of pest suppression tactics such as mating disruption, insecticides, and the sterile insect technique (SIT) can work together in an additive or synergistic fashion, with potential for multiple Allee effects at low population densities [21]. These combined and complex disruptive effects are able to overcome the rate of population increase.

The sterile insect technique (SIT) is a complementary technology to the existing IFP program [11]. It involves the mass rearing and release of sterile males which compete with the wild males to mate with wild females, reducing the number of successful matings and offspring, and thereby suppressing the wild population. [22,23]. The eradication of pink bollworm *Pectinophora gossypiella* was achieved over a large area in the USA through the combination of sterile insect releases, mating disruption, and transgenic cotton [24]. A pioneering sterile insect program was developed against codling moth in British Columbia, Canada, based on the extensive field and laboratory research of Proverbs et al. [25,26,27,28] and others [29,30]. The SIT has been deployed for nearly three decades against this species in that program, using weekly ground-based releases [31]. This early Canadian initiative to develop the SIT for codling moth [25] led to the development of a factory and long-standing release of moths into orchards for population suppression [31]. While this program started out with the ambitious target of eradication, the program was later moved to suppression [31]. There are many known biological and logistical challenges found in the cost-effective application of the SIT for codling moth control, reviewed in extensively by Thistlewood and Judd in 2019 [32]. This includes, but is not limited to, asynchrony in mating between sterile and wild populations due to weather and potentially aggregated populations, but also the requirement for already low population density to be effective. This is in contrast to tactics such as mating disruption and particularly insecticides which are effective at higher population densities.

Despite no known global mating incompatibility in this species [33], and that other factors including cost-effective rearing and release systems have been improved [23], codling moth SIT for pest management has been relatively slow to spread from Canada. Despite positive results, earlier attempts to develop codling moth SIT in South Africa, first a trial using Canadian insects [34] and later a domestic supply from a company, Entomon Technologies (Pty) Ltd (Stellenbosch, South Africa), in 2011, ended due to relaxation of residue requirements in export markets, low economies of scale in insect rearing, and belief by growers on the efficiency of insecticides [35]. The supply of sterile codling moth from Canada to New Zealand has provided an opportunity to demonstrate how the addition of SIT to already very low populations could synergize pheromone-based export apple IFP [11].

With very low codling moth densities already, we aimed to test the potential for the sterile insect technique (SIT) to supplement mating disruption and insecticides to achieve local eradication or at least major suppression of this pest. The goal was to demonstrate the potential for avoiding the need for methyl bromide or other fumigation altogether by establishing an area of low pest prevalence (ALPP) [36], or establishing a Pest-Free Area (PFA) [37], in the event of eradicating codling moth altogether. Suppression was defined as a significant reduction in codling moth populations. Eradication was defined consistent with the International Plant Protection Convention (IPPC) International Standards for Phytosanitary Measures (ISPM) 5 definition of eradication (“Application of phytosanitary measures to eliminate a pest from an area”). To be met, zero trap catches for two years under continuous treatment would be required, and then would enable removal of mating disruption with ongoing trapping and surveillance as evidence of an Area of Freedom. The main difference between an ALPP and a PFA is that the presence of the pest below a specified population level is accepted in an ALPP, whereas the pest is absent from a PFA. It should be noted that this research program was not operating under the IPPC definition of official control (the active enforcement of mandatory phytosanitary regulations and the application of mandatory phytosanitary procedures with the objective of eradication or containment of quarantine pests or for the management of regulated non-quarantine pests) [38]. We targeted an isolated rural sub-region in Central Hawke’s Bay, New Zealand, to test the combined effects of the SIT, mating disruption, and selective larvacides for reducing codling moth levels. The nature of large-scale experiments with hundreds of hectares of complex export orchards meant that not all variables could be controlled, since pest management decisions such as pre-emptive insecticides were the ambit of the particular orchard managers at the enterprise level. A related trapping study in host trees had provided local insights into non-orchard populations, which were present in the sub-region but comparatively sparse on the roadside apple trees or in gardens several kilometers away [15]. Peri-urban householder views about SIT were also examined in a related study [39].

## 2. Materials and Methods

### 2.1. Orchards

**Figure 2:**
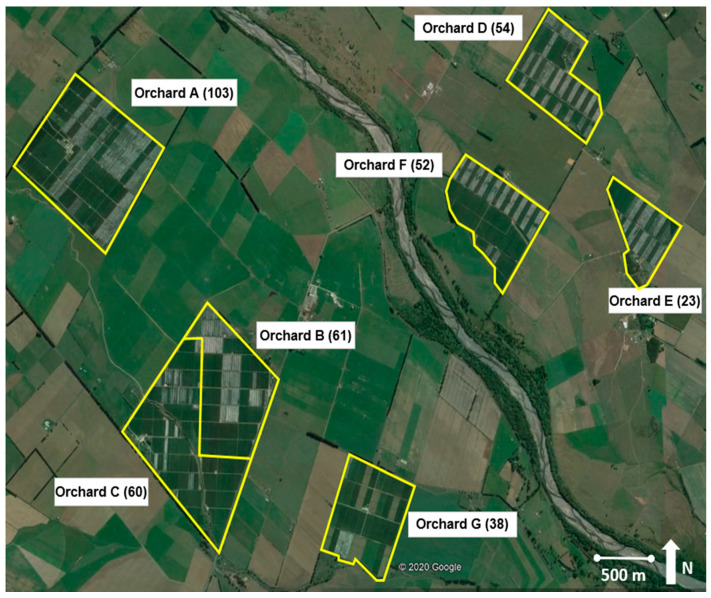
Map of the seven orchards with size (ha) in brackets involved in the pilot eradication program using sterile insect release, insecticides, and mating disruption surrounded by pastoral land. Map image courtesy of Google Earth (4 May 2020).

Six IFP orchards (total 353 ha), and one 38 ha BioGro™ New Zealand certified organic orchard, near the small settlement of Ongaonga in Central Hawke’s Bay, New Zealand (−39.885483° S, 176.468342° E) (Figure 1 and Figure 2), were involved in the program (Supplementary Table S1). The orchards were isolated from the main orchard district of the Heretaunga Plains surrounding Hastings, ~45 km away, and were isolated from each other by surrounding pastoral farms. There are no other apple orchards in this sub-region, though there are known backyard apple and walnut trees within 3 km of the orchards [15]. Two sets of data were used to test the effect of SIT release over time and across orchards, replicated against a common background of other tactics.

A replicated before and after control and impact experimental design with random initial allocation of SIT to ensure spatial replication was applied, using 23–103 ha orchards as replicates. For the first two years, SIT was only applied in orchards A, B, and C, with D, E, and F as controls. The analysis compared catches in the 2014/15 and 2015/16 seasons, where orchards A–C had sterile insects released and orchards D–F did not. Looking across time at the same locations, the before and after SIT periods had a duration of two years for each orchard (orchards A–F with two different starting years). Thus, SIT releases began at orchards A–C in 2014–2015 and expanded to another three orchards (D–F) in 2016–2017. The organic orchard (G) was included in 2017–2018. Results for longer periods of SIT were viewed graphically.

### 2.2. Sterile Insect Technique

#### 2.2.1. Insect Rearing, Shipment, and Quality Control

Mass-reared male and female sterile codling moths were provided by Okanagan-Kootenay Sterile Insect Release Program (OKSIR) in Osoyoos, British Columbia, Canada. They were irradiated as newly enclosed adults at 150 Gy in Petri dishes containing ~800 males and females at a ratio of ca. 1:1. Adult treatment at this dosage was expected to result in fully sterile females and a low level of F_1_-inherited sterility in wild crosses with males [40]. Insects were packaged and cool-shipped as per Carpenter et al. [34], and data loggers were co-shipped to confirm that the temperature remained between 0 and 2 °C during delivery. Quality control has continued to be an important area of investigation [41].

The following quality control tests were carried out to confirm the biological quality of the shipped insects as compared with insects that were not shipped. All quality control tests were conducted in environmentally controlled rooms (27 ± 0.5 °C, 16:8 (Light:Dark) photoperiod, 30% Relative Humidity) in New Zealand and Canada and were replicated for four shipments. 1. Flight ability: Three flight cylinders were produced by cutting polyvinyl chloride (PVC) pipe (16 cm diameter) to a height of 16 cm and were placed on smooth arborite board sealed with filler at the base to prevent light penetration. The inner cylinders were coated with talcum powder to prevent moths crawling out. Chilled (2 °C) male moths (n = 25) were placed on the table surface inside each of the flight cylinders. No females were present in the room for these experiments so that male flight propensity was not affected by sex pheromone presence. The number of moths remaining in each cylinder after a period of 24 and 48 h was recorded. 2. Longevity: Insects (n = 10 per sex) were placed individually in 30 mL plastic jars without water. Mortality was checked every 24 h to determine longevity of insects under stress. This was replicated concurrently (n = 10 per sex) with moistened dental rolls (i.e., with water). 3. Mating ability: Four 30 × 30 × 30 cm cages had 10 moth pairs placed in each. After 24 h, the females in two cages were dissected, and mating status concluded by the presence of a spermatophore in the bursa copulatrix. After 48 h, the females in the remaining two cages were assessed.

The shipping pathway involved three commercial flights as well as customs clearance and importation under NZ Ministry for Primary Industries approval (Permit No. 2015058400). There were 14–15 weekly shipments from November to March each season. Due to the OKSIR factory shutting down over the Christmas period, insects were not released for 2 weeks (weeks 52 and 1). Insect release numbers are reported in Supplementary Materials Table S2, with a mean of 2245 moths/ha/season. Those orchards with higher wild populations before the addition of SIT, particularly orchards E and G, had a much higher number of sterile insects released per hectare to meet the overflooding ratio required.

#### 2.2.2. Quality Control Testing of Insects Released from Unmanned Aerial Vehicle

In advance of beginning field releases of sterile insects by Unmanned Aerial Vehicle (UAV), a series of laboratory experiments were carried out to assess the biological quality of insects following release from the release device. Experiments were conducted at the Okanagan-Kootenay Sterile Insect Release Facility in Osoyoos, British Columbia, Canada, in environmentally controlled rooms (25 ± 0.5 °C, 16:8 (L:D) photoperiod, RH). A high-powered fan forcing air through a laminar flow setup simulated the effect of wind when the aircraft was moving at 50 km/h. Insects were released over a 5.6 min “flight” time, with the target release rate being 45 g insects/minute. Insect biological quality was assessed following 3 identical releases. The following quality control tests were carried out to confirm the biological quality of the UAV-released insects as compared with control insects: 1. Flight ability: As described in Section 2.2.1, chilled (2 °C) male moths (n = 20) were placed on the table surface inside each of the flight cylinders. The number of moths remaining in each cylinder after a period of 24 and 48 h was recorded. 2. Longevity: Insects (n = 10 per sex) were placed individually in 30 mL plastic jars without water in an environmentally controlled room (27 ± 0.2 °C 16:8 (L:D) photoperiod, 30% RH). Mortality was checked every 24 h to determine longevity of insects under stress. 3. Mating in cages: Members of each sex released from UAV and those not (n = 10 per treatment) were mated with “wild” non-irradiated insects. 10 pairs of each cross were put into 30 × 30 × 30 cm cages for 48 h and then their bursa copulatrix was dissected to determine mating status. 4. Mechanical damage: 10 moths of each sex for both UAV-released and control insects were examined for mechanical damage to antennae, wings, scales, and legs. Insects were killed by freezing and damage was visually estimated as percentage loss using a compound microscope.

#### 2.2.3. Field Release of Insects

Insect release rates on each property (Supplementary Table S2) were guided by calculating the target overflooding ratio (40:1) based on the cumulative wild population data from the previous season and the size of the orchard. In the early years of the program (2014–2015 and 2015–2016), insects were ground-released via a combination of mountain bikes and an all-terrain vehicle. In 2016–2017, the program began delivering insects using an Air Titan fixed-wing, unmanned aerial vehicle (UAV) flying at 70 km/h at a height of 40–50 m with a three-dimensional (3D)-printed radio-controlled release device attached underneath (Supplementary Video S1). Pre-programmed flights take ~10 min to deliver 20,000 moths across 100 ha. We operated the UAV under Part 101 of the New Zealand Civil Aviation Rules, which requires us to remain within line of sight of the aircraft. The variable island climate of New Zealand has posed challenges for release by UAV, with some flights grounded due to high winds. When this occurred, we either returned the next day or released moths by driving around the orchard tracks.

### 2.3. Monitoring Adult Codling Moths

All commercial export apple orchards in New Zealand use pheromone traps and report codling moth numbers each week [11]. Data from moth catch in traps for all seven orchards was available for 2–4 years before the start of sterile insect releases. Red delta traps made of Corflute™ plastic with a sticky base (19 by 18 cm) (Desire™, Etec Crop Solutions Ltd., Auckland, New Zealand) were baited with 1 mg codlemone (99% purity) rubber septa lures from commercial suppliers (Etec Crop Solutions Ltd., Auckland, New Zealand). This is the New Zealand apple industry standard, except where mating disruption is applied to orchards, where lures are loaded at 10 mg of codlemone to overcome the background concentration of codlemone coming from the mating disruption dispensers. Orchards D–G had 10 mg lures in all years. Orchards A–C lure use varied by location and year due to partial coverage of mating disruption, but by 2017, they only used 10 mg lures due to full mating disruption coverage. Whilst bi-sex lures containing codlemone and pear ester are commonly used overseas, these are not commercially available to growers in New Zealand, so codlemone alone is used. All traps were set at approximately 3 m in height on bamboo poles and were checked weekly for moths, and pheromone lures were changed every 6 weeks during the growing season from October to March. Trap density was set at 1 trap per 1–2 hectares, as prescribed for export orchards. Orchard G had a much lower trap density of 0.18–0.5/ha. The number of traps operated in each orchard each year is provided in Supplementary Table S1. Numbers of wild and sterile moths in traps were recorded separately. Sterile moths are marked internally by the addition of Calco-Red dye to the larval diet, making them discernable from wild insects in traps, as they are checked [42]. Due to low numbers of wild insects captured in pheromone traps, and the changing numbers of pheromone traps between seasons, wild catches were summarized as male moths caught per 10 pheromone traps per season. Recapture rates of sterile males were summarized as percentage of male moths released that were recaptured. Whilst moths released were both sexes, pheromone traps catch only males, so a 1:1 male female sex ratio was assumed. The achieved overflooding ratio was defined as the ratio of sterile males recaptured in traps to the number of wild males caught in traps.

### 2.4. Mating Disruption

Commercially available mating disruption dispensers (ISOMATE^®^4Play or ISOMATE^®^- C PLUS MD FLEX) were applied to orchards annually at 800 ties/ha. In orchards A–C in the two years preceding SIT release, the percentage coverage of mating disruption ranged from 51% to 60%. In the first three years of the SIT releases, in orchards A–C, coverage increased and ranged from 83% to 94%. For orchards D–G, there was 100% mating disruption coverage in the two years preceding the application of SIT, and this remained at 100% for the duration of the SIT pilot program. The percentage of each orchard covered by mating disruption in each year is shown in Supplementary Table S3.

### 2.5. Insecticides

The timing of the first insecticide application each season was based on the regional BIOFIX (first moth flight) + 100 degree-days (°C base 10), as indicated by male trapping. This is timed to coincide with the earliest regional egg hatch of the first generation of codling moth [43]. This first phenology-based selective larvicide application is a mandatory requirement in Ministry for Primary Industries ‘Codling Moth-Sensitive Market’ (CMSM) program. Thereafter, the ‘CMSM’ program requires that all insecticide use for codling moth control is based on pheromone trapping thresholds: 5 or more moths in a single trap in a one week OR average weekly catch in all traps of 2 or more moths OR an accumulation of 10 or more moths (or 5 or more moths from 1 January) caught in a single trap since the last insecticide spray. However, these thresholds define the minimum standard for ‘CMSM’ registered production sites, and additional applications of larvicides can be applied at any time (Table 1). For example, a codling moth spray was always applied in late December when shipments of sterile moths were suspended to provide additional crop protection. The insecticides used for codling moth control include: methoxyfenozide, chlorantraniloprole, indoxacarb, and codling moth granulosis virus (Supplementary Table S4).

### 2.6. Fruit Damage Assessments

Extensive checks of fruit were conducted in the orchard and packhouse, following the standard protocol on commercial export orchards. At each orchard, random samples of harvested fruit in bins were inspected in the field for pests by orchard staff performing quality control checks. The number of bins inspected varied amongst orchards. In addition, all fruit underwent grading by defect sorting machines and human inspections following strict export consignment protocols. Because there were no finds of codling moth in bins in the field or in the packhouse in any of the orchards, no analysis was carried out.

### 2.7. Data Analysis

The flight ability and longevity of shipped and non-shipped insects were compared by 2-sample *t*-test and differences were considered significant at an alpha value of 0.05. The proportion of mating of shipped and non-shipped insects were compared by a 2 proportions *Z*-test. UAV-released insects were compared to control insects by 2-sample *t*-test and differences were considered significant at an alpha value of 0.05. The proportion of mating of UAV-released and control insects were compared by a 2 proportions *Z*-test.

The average wild trap catch per trap per week was calculated for each orchard and season, using the data from the weeks observed in all orchards and seasons (weeks 49–52 and 1–10). Two sets of data were tested to investigate the effect of SIT release. The first compared catches at each orchard (A–F) in the two seasons before SIT release with the two seasons after SIT release, while the second compared catches in the 2014–2015 and 2015–2016 seasons, where orchards A–C had SIT released and orchards D–F did not. For both comparisons, the percentage coverage of mating disruption per orchard per season and the number of codling moth insecticides used per orchard per season were fitted before testing the effect of SIT.

The first comparison for an SIT effect compared with controls was tested using a Poisson generalized linear model with factors for orchard, SIT/no SIT and the orchard × SIT/no SIT interaction. Because the trap data were averages, they were less dispersed than a typical Poisson distribution, so a dispersion factor was estimated from the residual deviance and used in the analysis.

The second comparison for the same orchard changes used a Poisson generalized linear model with factors for season (2014–2015 or 2015–2016), SIT/no SIT, and orchard nested within SIT/no SIT. Again, the data were less dispersed than a typical Poisson distribution, so a dispersion factor was estimated from the residual deviance and used in the analysis. Because the orchard within SIT/no SIT effect was significant, this was followed up by fitting a Poisson generalized linear mixed model, with fixed effects for season and SIT/no SIT, and a random effect for orchard nested within SIT/no SIT. This tested whether the SIT/no SIT effect was significantly larger than the orchard to orchard variation. Analysis was carried out in Genstat version 20 [44]. The full dataset is available in Supplementary materials File S1. 

## 3. Results

### 3.1. Quality Control of Long-Distance Shipped Insects

Comparisons in quality between insects that had been shipped to New Zealand with insects that had not been shipped showed variable results, but overall, there was little evidence of a reduction in biological quality. For the number of insects flying out of the flight cylinders after 24 h, there was no significant difference between shipped (mean (M) = 16.83, standard deviation (SD) = 3.07) and non-shipped insects (M = 14.33, SD = 3.45); T = −1.88, *p* = 0.075. After 48 h, there remained no significant difference in the number of insects exiting the flight cylinders between shipped (M = 21, SD = 2.04) and non-shipped (M = 18.92, SD = 3.09) insects; T = −1.95, *p* = 0.07. For longevity of females without water, there was no significant difference between shipped (M = 10.7, SD = 1.7) and non-shipped insects (M = 10.95, SD = 4.43); T = −0.33, *p* = 0.744. For longevity of males without water, there was no significant difference between shipped (M = 7.45, SD = 2.55) and non-shipped insects (M = 7.88, SD = 1.34); T = −0.93, *p* = 0.36. For longevity of females with water, there was a significant difference between shipped (M = 15.40, SD = 3.03) and non-shipped insects (M = 13.40, SD = 4.67); T = 2.27, *p* = 0.026. For longevity of males with water, there was no significant difference between shipped (M = 15.38, SD = 2.82) and non-shipped insects (M = 14.23, SD = 4.75); T = 1.32, *p* = 0.193. After 24 h, mating was significantly lower (−0.23) in the shipped insects than the non-shipped insects (z = 3.0, *p* = 0.003). However, after 48 h, there was no difference in mating between the non-shipped insects and the shipped insects (z = 1.59, *p* = 0.11).

### 3.2. Quality Control of Insects Released from UAV Device

Comparisons in quality between insects that had been released from the UAV release device and control insects that had not been released from the UAV release device showed little evidence of a reduction in biological quality. There was no significant difference in the flight ability after 24 h of insects released from the UAV release device (M = 8.17, SD = 4.36) and the control insects (M = 8.00, SD = 4.36); T = −0.06, *p* = 0.957. There was no significant difference in the flight ability after 48 h of insects released from the UAV release device (M = 13.50, SD = 1.38) and the control insects (M = 13.33, SD = 2.08); T = −0.13, *p* = 0.912. There was no significant difference in the longevity in days of insects released from the UAV release device (M = 5.70, SD = 2.26) and the control insects (M = 5.83, SD = 1.98); T = 0.34, *p* = 0.731. There was no significant difference in the mating success of males released from UAV and control males (z = 1.46, *p* = 0.143). There was also no significant difference in the mating success of females released from UAV and control females as all insects successfully mated. There was rare loss of antennae (~4%) and legs (<1%). However, this occurred equally in the control and the UAV insects. These appendages are known to be occasionally lost in the factory ducting. No scale loss was observed in either control or UAV-released insects.

### 3.3. Field Trials

**Figure 3:**
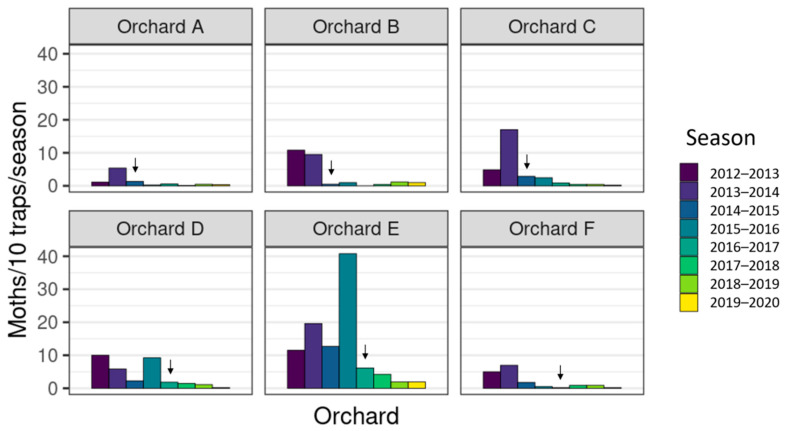
Season-long catch of wild male codling moths (*Cydia pomonella*) per 10 traps (1–2 traps/ha), in each of six integrated fruit production (IFP) apple orchards (total 353 ha), with increased mating disruption (Supplementary Table S3), over the past eight years. Arrows indicate the first year that sterile insects were released.

**Figure 4:**
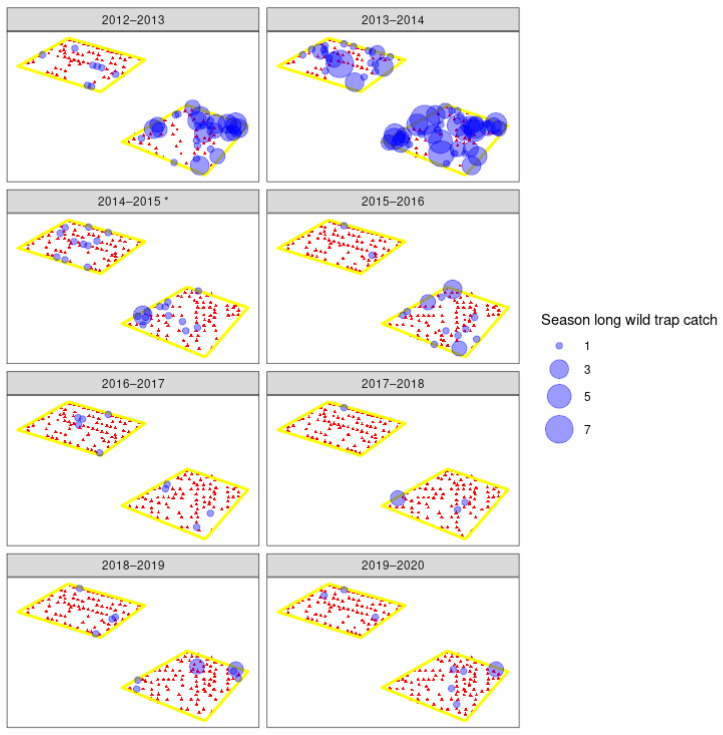
Spatial distribution of season-long wild male codling moth (*Cydia pomonella*) catches in traps in three integrated fruit production orchards (A–C) that are part of a pilot sterile insect release program. Orchards B and C share a border and are presented as one polygon (see Figure 2). Red triangles indicate pheromone traps that did not catch any wild moths, and the blue circles indicate the number of moths caught in that trap across the codling moth season for each year. Asterisk indicates the first year of sterile insect releases in those orchards.

**Figure 5:**
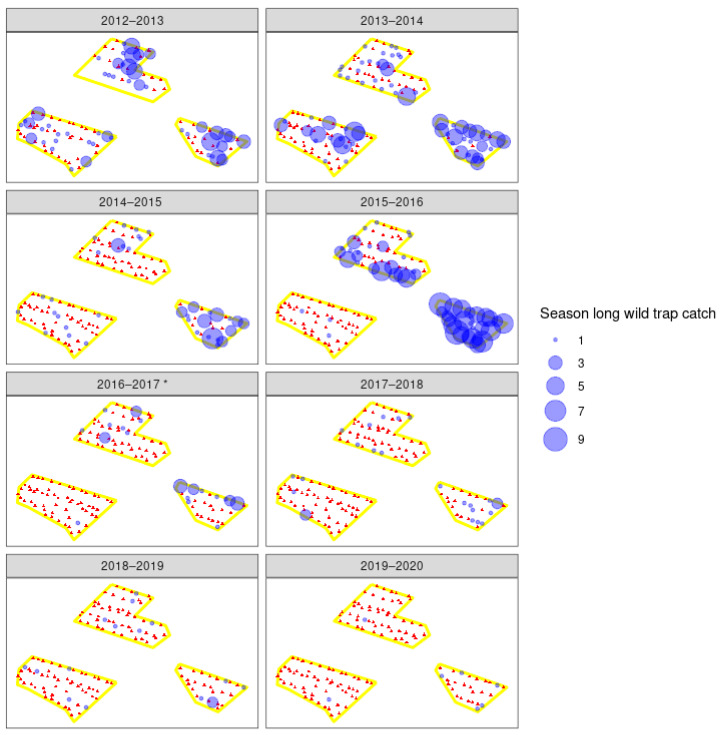
Spatial distribution of season-long wild male codling moth (*Cydia pomonella*) catches in traps in three integrated fruit production orchards (see Figure 2) (D–F) that are part of a pilot sterile insect release program. Red triangles indicate pheromone traps that did not catch any wild moths, and the blue circles indicate the number of moths caught in that trap across the codling moth season for each year. Asterisk indicates the first year of sterile insect releases in those orchards.

**Figure 6:**
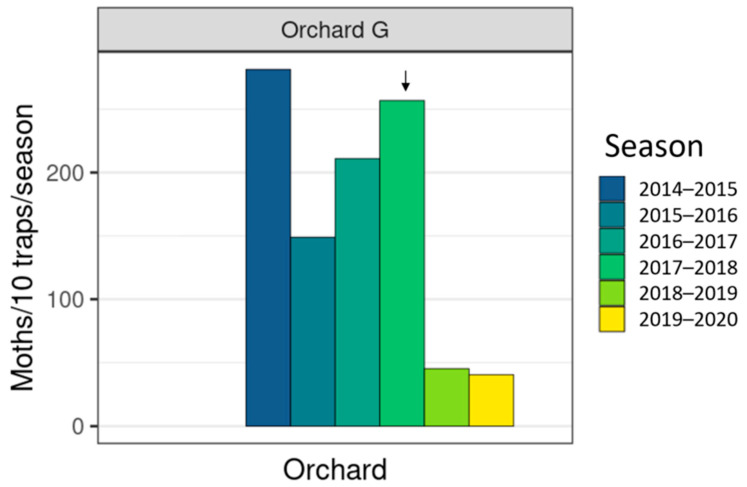
Season-long catch of wild male codling moths (*Cydia pomonella*) per 10 traps across 6 seasons (~2–3 traps/ha), in organic “Orchard G”, over the past six years. The arrow indicates the first year that sterile insects were released.

Male moth catches in traps in all seven orchards showed large reductions in catch after the introduction of the sterile insect technique, in combination with insecticides and mating disruption (Figure 3, Figure 4 and Figure 5). After the sixth season of releases in the 2019–2020 season, catches of wild codling moth at orchards A–C were 90–99% lower than in 2013–2014, the season before releases began, and only 7 wild moths were caught across 186 traps. Orchards D–F have had catches decrease by 67–97% since 2015–2016, the year before releases began, and only 7 wild moths were caught across 136 traps in the 2019–2020 season. The organic orchard G had an 81% reduction in moth catch from 2016 to 2017, starting from much higher initial populations than orchards A–F, and 77 wild moth were caught across 19 traps in the 2019–2020 season (Figure 6). There were large decreases in male moth catches in the first year of sterile insect releases at all 6 IPM orchards (67% to 95%), but not at orchard G, where a 22% increase in catch was observed. When comparing catches at each orchard (A–F) in the two seasons before SIT release with the two seasons after SIT insect release, the model indicated a significant decline in wild trap catches after the introduction of SIT (F = 27.7 on 1 and 12 df, *p* < 0.001). This did not differ significantly between orchards (interaction F = 0.2 on 5 and 12 df, *p* = 0.972). The SIT effect was similar for both orchards A–C (change in 2014–2015) and D–F (change in 2016–2017) (SIT × A–C vs. D–F interaction, F = 0.2 on 1 and 12 df, *p* = 0.661). Fitting the number of CM insecticides used and the percentage coverage of mating disruption per orchard per season to the model before testing the effect of SIT did not change the conclusion that there was a significant decline in wild trap catches after the introduction of SIT.

When comparing catches in the 2014–2015 and 2015–2016 seasons, where orchards A–C had SIT released and orchards D–F did not, the average wild trap catch was lower in the SIT orchards than the non-SIT orchards (F = 47.2 on 1 and 5 df, *p* < 0.001), but the effect varied from orchard to orchard (orchard nested within SIT/no SIT F = 14.2 on 4 and 5 df, *p* = 0.006). The generalized linear mixed model indicated that the SIT/no SIT effect was not significantly larger than the orchard nested within SIT/no SIT variation (F = 3.1 on 1 and 4 df, *p* = 0.147). The difference between SIT and non-SIT orchards may be randomly affected by other management differences. Fitting the number of CM insecticides used and the percentage coverage of mating disruption per orchard per season before testing the effect of SIT made the SIT effect in the GLM not significant (F = 4.2 on 1 and 3 df, *p* = 0.132). The difference in effect between orchards was also reduced, but not as much. In the mixed model, the SIT/no SIT effect was not significantly larger than the orchard nested within SIT/no SIT variation (F = 2.8 on 1 and 6 df, *p* = 0.138).

**Figure 7:**
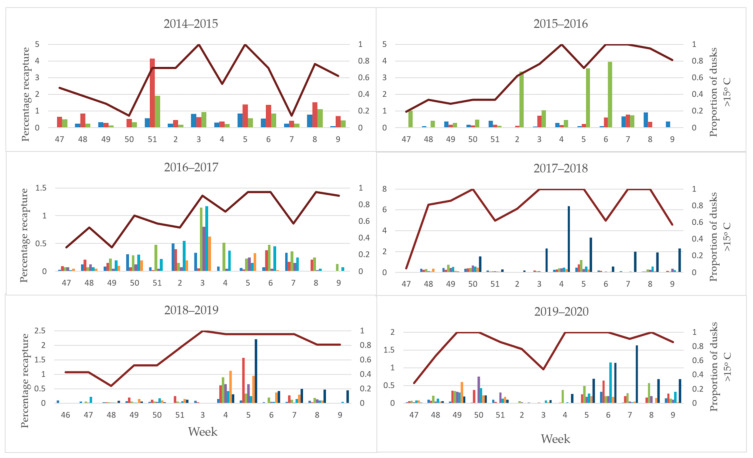
Percentage of sterile male codling moths (*Cydia pomonella*) recaptured per week across the 7 orchards (1 trap/ 1–2 ha), note variation in Y scale (left Y axis). The maroon line indicates the proportion of dusk hours that were above the codling moth flight threshold of 15 °C (right Y axis).

**Figure 8:**
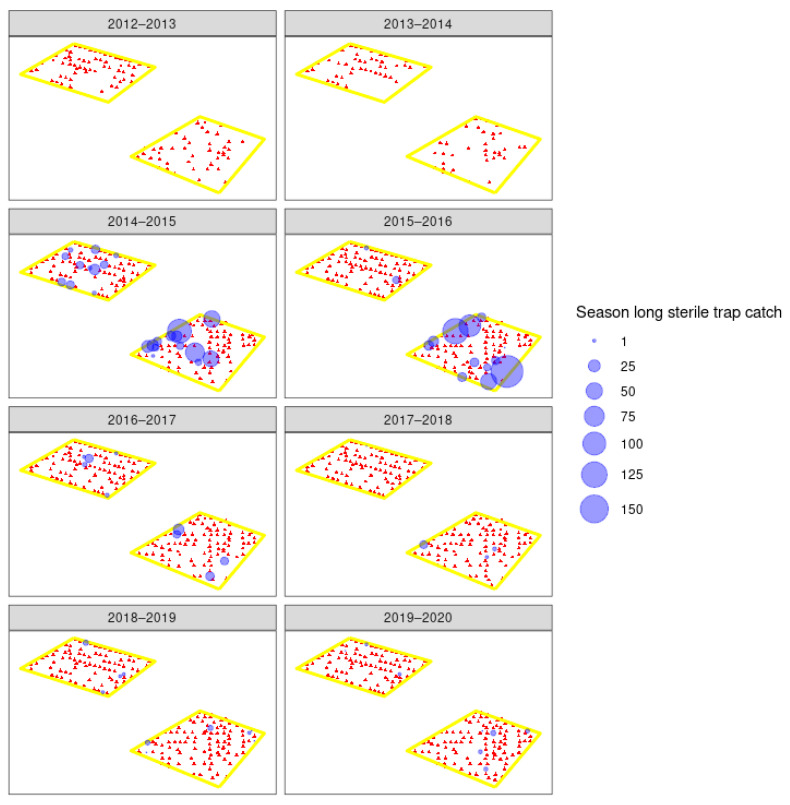
Spatial distribution of sterile male codling moth (*Cydia pomonella*) recaptured following release in three apple orchards (A–C) for six years. Orchards B and C share a border and are presented as one polygon (see Figure 2). Red triangles indicate pheromone traps that did not catch any sterile moths, and the blue circles indicate the number of sterile moth recaptured across the codling moth season.

**Figure 9:**
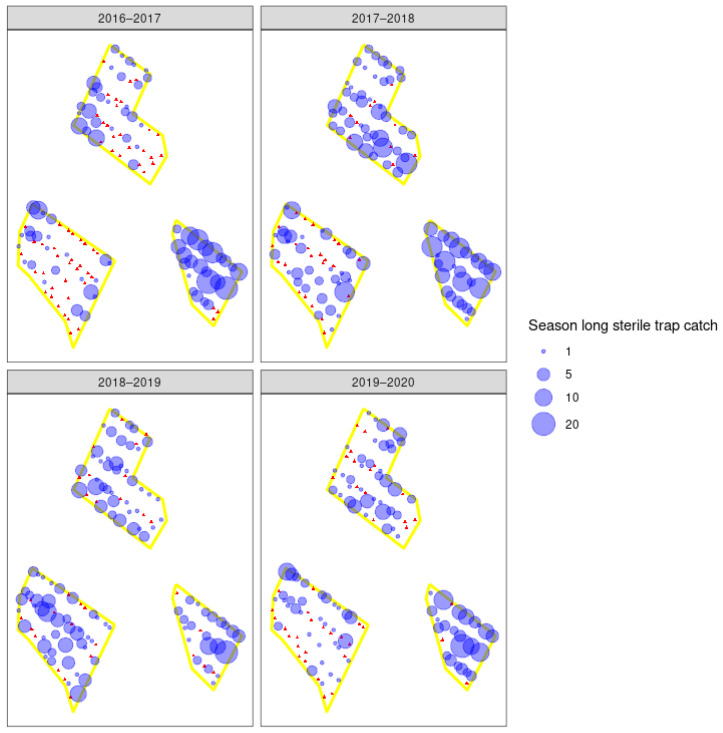
Spatial distribution of sterile male codling moth (*Cydia pomonella*) recaptured following release in three apple orchards (see Figure 2) (D–F) for six years. Red triangles indicate pheromone traps that did not catch any sterile moths, and the blue circles indicate the number of sterile moth recaptured across the codling moth season.

The overflooding ratio, defined as the ratio of number of sterile males recaptured to the number of wild males captured varied from as low as 3:1 sterile to wild males and as high as 306:1. This fluctuated heavily between orchards and years (Table 2), and did not increase over the years. However, with such low numbers of wild moths, this is perhaps not surprising. The number of sterile insects recaptured in each orchard for each year is shown in Figure 7, Figure 8 and Figure 9. From the 2016–2017 season to 2020, releases were made by UAV, and recapture was higher in the early years of 2014–2015 where insects were released by ground methods. Specific results of this work and further work with hexacopter releases will be presented elsewhere (Lo et al. in prep). Weekly recapture rates of sterile insects were found to follow the dusk temperatures, with recaptures in weeks 47–50 often low due to lower spring temperatures (Figure 7). Recapture rates of sterile insects were higher in orchard G than in orchards A–F. This is likely due to extensive tree removal due to disease reducing the efficacy of mating disruption.

## 4. Discussion

We undertook a large-scale and complex long-term study to test suppression and eradication technologies for the codling moth at the landscape scale in an isolated group of orchards in New Zealand. Overall, the New Zealand pilot codling moth SIT release program faced technically difficult challenges due to the complexity of weekly trans-national shipments and border clearance, to the release and distribution over hundreds of hectares using live adult moths, all with a target of <40 h from factory to release. Fortunately, no insect performance issues were found to arise as a result of the long-distance shipping required to transport the insects to New Zealand, corroborating successful long-distance shipping to South Africa from Canada [34]. Despite these challenges, the added benefits of SIT to New Zealand’s existing ‘systems-based’ approach to production are obvious in the reduced pest densities achieved, and the stage is set to expand the scale, should growers and exporters pursue the required investment. Future New Zealand programs could involve partial rearing from imported eggs, or complete year-round production as in Canada. Use of SIT, in combination with New Zealand’s existing on-orchard controls for codling moth, could provide a position to reduce phytosanitary justification for mandatory postharvest disinfestation with unwanted fumigants.

Eradication was not achieved across the comparatively isolated region, but a very high level of codling moth suppression was achieved at individual orchards after the introduction of sterile moths in combination with mating disruption and larvicides. The insect is scarcer, but the regional populations have not been eradicated, despite six years of on-orchard releases. It is clear that efforts are needed off-orchard also, such as systematic area-wide host removal [15]. Consultation with the peri-urban public indicated a willingness to cooperate over managing backyard trees [39], but a specific program would be needed for comprehensive host removal. For the conditions of the IPPC ISPM 5 definition of eradication to be met, zero trap catches for two years under continuous treatment would have been required. This would enable removal of mating disruption with ongoing trapping and surveillance as evidence of an Area of Freedom. Failure to eradicate would require the inputs required for market access to continue, as now.

The challenges of eradicating any insect are well known [45], but specific knowledge of an attempt at codling moth eradication is otherwise only available from the OKSIR program that began in 1992 [32]. That program has operated in three modes of: (1) Rapid eradication (1992–1996), (2) slow eradication (1996–1999), and (3) area-wide management (since 2000), when eradication was deemed not economically and socially feasible [32]. Program goals were changed to reduce insecticide use and to reduce damage from codling moths to a low level of 0.2% damage in 90% of orchards. The economics of the OKSIR program have been justified by environmental savings with reduced insecticides [32]. However, the investment in a potential area-wide New Zealand SIT program would likely be justified by achieving access to high-value markets, i.e., economic gain.

Haphazard re-infestation of the orchards could be occurring via dispersal from neighboring backyard walnut, apple, and pear trees [15], to maintain or re-establish the pest population, as was the case in British Columbia [32]. Given what has been learned about on- and off-orchard populations in the area [15], and that no special program of suppression was provided for peri-urban or other host trees in the sub-region, lack of extinction is unsurprising. The late entry and higher starting populations of the organic orchard could also have provided further pressure on the program. The way forward needs to aim to reduce the remaining host trees as far as possible, following the imperative to tackle sources on an area-wide basis, if it is to demonstrate the requirements for Area of Freedom. However, our results point the way for future attempts to eradicate codling moth, including the need for community involvement, which is fortunately favorable to using sterile insects [39]. If orchards continue to maintain ultra-low populations for export, this is still a major benefit in the interim. Ongoing pest management would be expected to have higher inputs of suppression tactics over time, while in the case of eradication, only trapping is needed for proof of freedom.

### 4.1. Wild Moth Trends

We recognize the limitations of conducting an area-wide SIT program on export orchards that operate under strict phytosanitary regulations. In order to meet phytosanitary requirements, phenology and threshold-based sprays had to be applied, and mating disruption was highly recommended to the orchards. The combined use of mating disruption and insecticides, as well as the sterile technique, meant that there was no true control, i.e., investigation of the impact of sterile insect technique alone versus no codling moth control, versus individual tactics. However, when comparing catches before SIT release with the two seasons after SIT release, there was a significant decline in wild trap catches after the introduction of SIT, even after investigating the impact of additional control measures. It must also be noted that mating disruption in orchards A–C did increase from 50% coverage over the same period that SIT was applied, to 100% coverage. However, in orchards D–G, 100% mating disruption was in place in the two years preceding SIT, yet wild populations still dropped significantly following SIT releases beginning. When comparing the first two seasons (2014–2015 and 2015–2016) where SIT was applied only in orchards A, B, and C, with D, E, and F as putative controls, there was evidence that the lower trap catch in SIT-treated orchards when compared to non-SIT orchards may be due to other management differences, such as the number of insecticides and percentage coverage of mating disruption. This weakness in controlling the number of insecticides in the putative “control” orchards in the same year is fully acknowledged, leaving us with heavier reliance on the evidence of impact at each of the individual orchards when the full program of interventions was assembled (noted by adding SIT to 100% of coverage under mating disruption with larvicides). Although the populations all reduced, the initial dramatic drops in wild moth catch were not particularly well correlated with sterile insect recapture rates, which is another weakness in our data. The comparatively low efficiency of sex pheromone traps in the presence of mating disruption has hampered our assessment, but still enabled detection of some male moths at each orchard in each year. Comparatively poor performance in New Zealand of female codling moth attractants developed elsewhere [46,47,48] has hindered efforts to deliver a cost-effective commercial female lure thus far. However, improvements to tools for assessment of low populations are sought.

Multiple-tactic programs using sterile insects in combination with insecticides or mating disruption have been shown in the past to be effective at reducing codling moth populations, albeit from significantly higher starting populations than those described here [49].

At a radiation dose of 150 Gy, female codling moths are fully sterile, with a small amount of inherited sterility observed along the male line, leading to extinction at F_2_ [40]. However, a drop in wild male moth catch would not be expected until the following year (one generation) as the impact of lower fecundity and fertility was realized in a proportion of mated insects. However, we observed significant drops in wild male catch in the first season that sterile insects were released. It is highly probable that unmated sterile females were calling and competing with traps, resulting in lower wild male catch in pheromone traps, as reported in another species [50]. It also may be that the thousands of sterile females released were acting as sperm sinks for wild males, which can mate more than once. The percentage cover of mating disruption treatment was also increasing in some orchards at this time, which would have had an impact on wild male catch, though this cannot explain the dramatic reduction observed.

Not all combinations of tactics have equal outcomes [21]. In particular, because both mating disruption and SIT operate with inverse density-dependence and become more efficient at low density, they are potentially synergistic in combination [45] with potential for multiple Allee effects, including mate location effects [51]. Thus two of the population management tactics used in the program are expected to act in synergy, as shown by population modelers [52], while larvicides are expected to be additive, according to field trials [53]. These combined, but complex, disruptive effects were able to overcome the rate of population increase, but only on the orchards. Individual isolated untreated host trees can obviously support a population, while even a low density of trees can certainly expand the colonized area greatly [15]. Our ability to actually record changes in population growth rates is limited by the tools we have at present, which are failing to represent low insect density, especially in the presence of mating disruption. Fruit damage is commonly used to measure efficacy of a control tactic [32]. However, already ultra-low levels of wild codling moth (0.73 moths per ha in 2013–2014 season) meant that no fruit damage was found on investigation of thousands of bins of apples, even before the SIT program began.

The sex pheromone-baited traps for males comprise a sensitive detection system for codling moth, and are used routinely in IPM, although there is some complexity in interpreting their sensitivity. One of the most difficult problems faced by the program as it has evolved is the threshold of detection for sex pheromone traps. Does one moth in a trap in a season constitute the symptom of a breeding population, and are we able to identify hotspots based on trapping information? One recent study in Michigan provides a surprisingly high estimated trap efficiency [54], where a catch of 1 male translates to approximately 4.7 males and 4.7 females present per hectare. However, this trial was carried out in orchards not under mating disruption, whereas all orchards in this pilot were eventually under full mating disruption. It has been suggested that the proportion catch of the 10 mg lure under mating disruption is about the same as the 1 mg lure that is not under mating disruption, but the big difference is that the sampling area is reduced by ~50%, thus 1 male caught potentially equates to 9–10 pairs per hectare (Larry Gut pers. comm.).

### 4.2. Sterile Moth Release and Recapture

Achieving the target sterile to wild overflooding ratios in each orchard is essential to the success of an SIT program. The inability to achieve overflooding ratios at many sites was considered to be a major part of the reason for the slow progress of the eradication program in Canada [32]. In many established SIT programs, target overflooding ratios are dynamic, and fluctuate, sometimes even daily, in response to changes in wild population catch, be that spatially or temporally [32]. However, due to the extremely low wild population of codling moths in these orchards, and the lack of any spatial or temporal trends visible in wild catch, particularly in the latter part of the program, a flat overflooding ratio was selected for each orchard each year, based on the catch of the previous season, and the insects available.

The overflooding ratio of sterile to wild insects recaptured varied extensively from season to season, and recapture rates indicated that we dipped below the goal of 40:1 overflooding ratio in some seasons. Of course, there would have been areas of low male density in the day(s) following releases (depending on weather and other factors), even though the weekly records showed relative uniformity in catch distribution.

The presence of mating disruption is likely to have been a confounding factor in sterile recapture, as previous research with sterile insects has shown [55]. The low recapture rate compared with other countries [55] may be partly explained by the flight threshold of codling moth, which is 15 °C [56], as dusk temperatures in New Zealand often do not hit this. The release method is also likely to have had an effect on sterile insect recapture, and further detail of this work will be discussed elsewhere (Lo et al., under review).

Since 2015, this program has been able to adapt from ground release to use of unmanned aerial vehicles to release sterile insects. Early in the program, treatment area was restricted by the time taken to release insects from the ground. Fortunately, the field program was quickly able to adopt an UAV and develop a prototype 3D-printed release device to increase the practicality of rapid field release and distribution. The current expansion would not have been possible without taking to the air, and similar SIT (and biological control) programs have reaped similar benefits from aerial delivery of insects [57]. Using UAVs in the variable climactic conditions in New Zealand has not been without challenges, with wind or rain occasionally forcing a delay or reversion to ground release. Related research on aerial and ground release systems for codling moth indicated some issues with altitude of moth release (Esch et al., in prep), as insects released too high tended to drift away from the target area. New release systems require evaluation for performance, but aerial release would appear to offer many advantages for improved quality and competitiveness, particularly for Lepidoptera, where there has been a system of dedicated fixed wing aircraft for a long time, in the case of the pink bollworm eradication program [58]. The use of 3D printing for release devices offers exciting prospects for sharing low-cost, high technology solutions, and the computer-aided design (CAD) plans can be shared electronically for local printing. An optimal system involving swarms of drones can even be envisaged for the future, depending on local air control regulations.

### 4.3. Future Prospects of an Area-Wide Program in NZ

The next step in the expansion of the program is to seek investors and consider the appropriate business model for New Zealand. Other attempts at establishing codling moth SIT programs, such as that in South Africa, have failed not because of a lack of biological success, rather because of a lack of uptake by growers to make it viable, mostly due to relaxation of residue requirements for their targeted export markets, and a preference for insecticides [35]. The potential economic gain of New Zealand apples achieving market access without postharvest treatment is vast. Another successful commercial SIT enterprise, XSIT in South Africa, is in a similar market access situation and is funded by citrus growers and exporters [59,60], which has the advantage of a feedback loop to seeking continuous improvement in insect quality.

During the six years of this pilot study, a major apple exporter has funded a small expansion of the SIT program to the main growing district of the Heretaunga Plains, but winter rearing capacity in Canada is ultimately limiting. There are significant costs involved with trans-national shipping of insects, and logistics has become much more complex in 2020 with the cancellation of direct Vancouver–Auckland flights. The potential for shipment of eggs with modular rear-out and irradiation facilities is being investigated. Recommendations from the research team exhort the consideration of area-wide principles in the development of a deployment plan for insects [61], highlight the need to ensure community engagement on a wider program basis [39], and propose further detailed scoping of low-cost mass rearing, including the identification of commercial investors and beneficiaries to take SIT forward.

## 5. Conclusions

In a six-year pilot codling moth sterile insect release program, in combination with New Zealand’s existing on-orchard controls for codling moth, orchards have seen significant reductions in wild male catch in traps ranging from 90% to 99%. Catches at three other IFP orchards (129 ha) decreased by 67–97% over four years of sterile insect releases. The pilot program had the complexity of trans-national shipment, release and distribution over hundreds of hectares using live adult moths, all with a target of <40 h from factory to release, but the use of unmanned aerial vehicles allowed for the efficient delivery of insects and wider expansion of the pilot. The orchards have been able to achieve ultra-low populations of codling moth required for export, which is a major benefit, even if eradication was not achieved. Industry support and expansion in area treated from the original 200 ha is the best evidence of success so far. In the current program, scale would ultimately be limited due to shipping costs, but currently, the program is far from the limits and expansion is possible.

## Figures and Tables

**Table 1:** Number of codling moth (*Cydia pomonella*) insecticides applied in each of the seven orchards involved in the pilot codling moth eradication program. Numbers of larvicidal insecticides sometimes vary between orchard subdivisions within large orchards and are at the discretion of orchardists, with the minimum recommendation to cover the two weeks in December without SIT.

Orchard Number	Number of Codling Moth Insecticides Applied							
	2012–2013	2013–2014	2014–2015	2015–2016	2016–2017	2017–2018	2018–2019	2019–2020
A	1	1–2	1	2	3–4	2	3	3
B	1	1	2	3–5	1–2	3	3	4
C	1	1–2	1–2	3–4	2–4	3	3	4
D	2	3–4	3–4	2–5	4–5	3	3	4
E	2	4	3	5	5–6	3	3	4
F	1	1–2	1–2	3–4	2–4	3	3	4
G	NA	NA	NA	NA	NA	6	4	3

**Table 2:** The ratio of sterile male codling moths (*Cydia pomonella*) to one wild male caught per year across the 7 orchards (1 trap/1–2 ha).

Orchard	2014–2015	2015–2016	2016–2017	2017–2018	2018–2019	2019–2020
Orchard A	50	178	32	267	23	29
Orchard B	306	46	100	84	30	28
Orchard C	63	83	64	115	64	157
Orchard D			8	17	17	90
Orchard E			10	14	15	24
Orchard F			63	19	26	71
Orchard G				3	5	7

## Supplementary Materials

The following are available online at https://www.mdpi.com/2075-4450/11/12/837/s1. Video S1: https://youtu.be/H8-TreEhqEk. Table S1: Area and number of pheromone traps in each of the seven orchards involved in the pilot codling moth (*Cydia pomonella*) eradication program, in Central Hawke’s Bay. Table S2: Number of sterile male and female (1:1) codling moths (*Cydia pomonella*) insects released per hectare per season from 2014–2015 until 2019–2020 in the seven orchards involved in the pilot codling moth eradication program. Table S3: Percentage coverage of mating disruption in each of the seven orchards involved in the pilot codling moth (*Cydia pomonella*) eradication program, which increased following advice from the researchers. Table S4: Number and active ingredient of codling moth (*Cydia pomonella*) larvicidal insecticides applied in each of the seven orchards involved in the pilot codling moth eradication program. File S1: All raw data from the field trials.
