# Supplementary material for: Combined Effects of Mating Disruption, Insecticides, and the Sterile Insect Technique on Cydia pomonella in New Zealand
Source: Insects. 2020 Nov 27;11(12):837. doi: 10.3390/insects11120837 (PMC7759808; doi:10.3390/insects11120837)
Supplement: Supplementary file 1 [file insects-11-00837-s001.zip › insects-990255-supplementary/insects-990255-supple-conversion/Supplementary materials Tables 1-4.docx]

**Video S1. https://youtu.be/H8-TreEhqEk**

**Table S1.** Area and number of pheromone traps in each of the seven orchards involved in the pilot codling moth, (*Cydia pomonella*) eradication program, in Central Hawke’s Bay.

| **Orchard** | **Area (ha)** | **Number of Pheromone Traps** | | | | | | | |
| --- | --- | --- | --- | --- | --- | --- | --- | --- | --- |
|  |  | **2012–2013** | **2013–2014** | **2014–2015** | **2015–2016** | **2016–2017** | **2017–2018** | **2018–2019** | **2019–2020** |
| **A** | 103 | 70 | 65 | 82 | 81 | 85 | 85 | 85 | 85 |
| B | 61 | 37 | 40 | 41 | 41 | 47 | 47 | 51 | 51 |
| C | 60 | 16 | 20 | 45 | 49 | 45 | 46 | 46 | 50 |
| D | 54 | 33 | 53 | 53 | 53 | 54 | 54 | 54 | 54 |
| E | 23 | 26 | 26 | 26 | 26 | 26 | 26 | 26 | 26 |
| F | 52 | 50 | 56 | 56 | 56 | 56 | 56 | 56 | 56 |
| G | 38 | n/a | n/a | 7 | 7 | 10 | 19 | 19 | 19 |

**Table S2.** Number of sterile male and female (1:1) codling moths (*Cydia pomonella*) insects released per hectare per season from 2014–2015 until 2019–2020 in the seven orchards involved in the pilot codling moth eradication program. .

| **Orchard** | **Number of Sterile Insects Released/ha** | | | | | |
| --- | --- | --- | --- | --- | --- | --- |
|  | **2014–2015** | **2015–2016** | **2016–2017** | **2017–2018** | **2018–2019** | **2019–2020** |
| A | 2221 | 2563 | 1973 | 2470 | 2423 | 2004 |
| B | 2557 | 2951 | 2230 | 2387 | 4407 | 3095 |
| C | 2253 | 2600 | 2267 | 2427 | 4480 | 3147 |
| D | 0 | 0 | 1926 | 2252 | 2133 | 1748 |
| E | 0 | 0 | 4870 | 4522 | 4522 | 4104 |
| F | 0 | 0 | 2000 | 2308 | 2000 | 1815 |
| G | 0 | 0 | 0 | 3558 | 4379 | 3621 |

**Table S3.** Percentage coverage of mating disruption in each of the seven orchards involved in the pilot codling moth (*Cydia pomonella*) eradication program, which increased following advice from the researchers.

| **Orchard** | **Percentage of Orchard Covered by Pheromone-Based Mating Disruption** | | | | | | | |
| --- | --- | --- | --- | --- | --- | --- | --- | --- |
|  | **2012–2013** | **2013–2014** | **2014–2015** | **2015–2016** | **2016–2017** | **2017–2018** | **2018–2019** | **2019–2020** |
| A | 57 | 54 | 83 | 94 | 92 | 100 | 100 | 100 |
| B | 51 | 60 | 93 | 93 | 87 | 100 | 100 | 100 |
| C | 52 | 52 | 89 | 86 | 85 | 100 | 100 | 100 |
| D |  |  | 100 | 100 | 100 | 100 | 100 | 100 |
| E |  |  | 100 | 100 | 100 | 100 | 100 | 100 |
| F |  |  | 100 | 100 | 100 | 100 | 100 | 100 |
| G |  |  | 100 | 100 | 100 | 100 | 100 | 100 |

**Table S4.** Number and active ingredient of codling moth (*Cydia pomonella*) larvicidal insecticides applied in each of the seven orchards involved in the pilot codling moth eradication program. Numbers of insecticides sometimes vary between orchard subdivisions within large orchards (range shown) and are at the discretion of orchardists, with the minimum recommendation to cover the two weeks in December without SIT, although most usage was higher, driven by a desire for achieving market access.

| Orchard | Season | Methoxy  -Fenozide | Chloran-Traniliprole | Abamectin  + Chlorantraniliprole | Granulosis  Virus | Indoxa-  Carb | Lufen-  Eron |
| --- | --- | --- | --- | --- | --- | --- | --- |
| A | **2012–2013** | 1 |  |  |  |  | 1 |
|  | **2013–2014** | 2 | 1 |  |  |  |  |
|  | **2014–2015** | 1 |  |  |  |  |  |
|  | **2015–2016** | 1 |  | 1 |  |  |  |
|  | **2016–2017** | 1 | 1-2 | 0-1 | 0-1 |  |  |
|  | **2017–2018** | 1 | 1 |  |  |  |  |
|  | **2018–2019** |  | 1 | 1 |  | 1 |  |
|  | **2019–2020** |  | 1 | 1 |  | 1 |  |
| B | **2012–2013** | 1 |  |  |  |  |  |
|  | **2013–2014** | 1 |  |  |  |  |  |
|  | **2014–2015** | 1 | 0-1 | 0-1 |  |  |  |
|  | **2015–2016** | 1 |  | 1-2 | 0-1 | 1 |  |
|  | **2016–2017** | 1 |  | 1 |  |  |  |
|  | **2017–2018** | 1 | 1 | 1 |  |  |  |
|  | **2018–2019** | 1 | 1 | 1 |  |  |  |
|  | **2019–2020** | 1 | 1 | 1 |  | 1 |  |
| C | **2012–2013** | 1 |  |  |  |  |  |
|  | **2013–2014** | 1 |  | 0-1 |  |  |  |
|  | **2014–2015** | 1 |  | 1 |  |  |  |
|  | **2015–2016** | 1 |  | 0-1 | 1 | 1 |  |
|  | **2016–2017** | 1 | 0-1 |  | 0-1 | 1 |  |
|  | **2017–2018** | 1 | 1 | 1 |  |  |  |
|  | **2018–2019** | 1 | 1 | 1 |  |  |  |
|  | **2019–2020** | 1 | 1 | 1 |  | 1 |  |
| D | **2012–2013** | 1 | 0-1 |  |  | 0-1 |  |
|  | **2013–2014** | 1 | 1 | 1 | 0-1 |  |  |
|  | **2014–2015** | 1 |  | 0-1 |  | 1 |  |
|  | **2015–2016** | 1 | 0-1 | 0-1 | 0-1 |  |  |
|  | **2016–2017** | 1 | 1-2 | 1 |  | 1 |  |
|  | **2017–2018** | 1 | 1 | 1 |  |  |  |
|  | **2018–2019** | 1 | 1 | 1 |  |  |  |
|  | **2019–2020** | 1 | 1 | 1 |  | 1 |  |
| E | **2012–2013** | 1 | 1 |  |  |  |  |
|  | **2013–2014** | 1 | 2 | 1 |  |  |  |
|  | **2014–2015** | 1 | 1 | 1 |  |  |  |
|  | **2015–2016** | 1 |  | 2 | 1 | 1 |  |
|  | **2016–2017** | 1 | 2 | 1 | 1 | 1 |  |
|  | **2017–2018** | 1 | 1 | 1 |  |  |  |
|  | **2018–2019** | 1 | 1 | 1 |  |  |  |
|  | **2019–2020** | 1 | 1 | 1 |  | 1 |  |
| F | **2012–2013** | 1 |  |  |  |  |  |
|  | **2013–2014** | 1 |  | 0-1 |  |  |  |
|  | **2014–2015** | 1 |  | 1 |  |  |  |
|  | **2015–2016** | 1 |  | 0-1 | 1 | 1 |  |
|  | **2016–2017** | 1 | 0-1 |  | 0-1 | 1 |  |
|  | **2017–2018** | 1 | 1 | 1 |  |  |  |
|  | **2018–2019** | 1 | 1 | 1 |  | 1 |  |
|  | **2019–2020** | 1 | 1 | 1 |  | 1 |  |
| G | **2017–2018** |  |  |  | 6 |  |  |
|  | **2018–2019** |  |  |  | 4 |  |  |
|  | **2019–2020** |  |  |  | 3 |  |  |
